# A computational method for estimating trunk muscle activations during gait using lower extremity muscle synergies

**DOI:** 10.3389/fbioe.2022.964359

**Published:** 2022-12-13

**Authors:** Geng Li, Di Ao, Marleny M. Vega, Mohammad S. Shourijeh, Payam Zandiyeh, Shuo-Hsiu Chang, Valerae O. Lewis, Nicholas J. Dunbar, Ata Babazadeh-Naseri, Andrew J. Baines, Benjamin J. Fregly

**Affiliations:** ^1^ Rice Computational Neuromechanics Laboratory, Department of Mechanical Engineering, Rice University, Houston, TX, United States; ^2^ Biomotion Laboratory, Department of Orthopaedic Surgery, McGovern Medical School at the University of Texas Health Science Center at Houston, Houston, TX, United States; ^3^ Department of Physical Medicine and Rehabilitation, McGovern Medical School at the University of Texas Health Science Center at Houston, Houston, TX, United States; ^4^ Neurorecovery Research Center, TIRR Memorial Hermann, Houston, TX, United States; ^5^ Department of Orthopaedic Oncology, University of Texas MD Anderson Cancer Center, Houston, TX, United States

**Keywords:** trunk muscle activations, muscle synergies, estimation of unmeasured muscle activations, personalized musculoskeletal model, EMG-driven musculoskeletal model, pelvic sarcoma

## Abstract

One of the surgical treatments for pelvic sarcoma is the restoration of hip function with a custom pelvic prosthesis after cancerous tumor removal. The orthopedic oncologist and orthopedic implant company must make numerous often subjective decisions regarding the design of the pelvic surgery and custom pelvic prosthesis. Using personalized musculoskeletal computer models to predict post-surgery walking function and custom pelvic prosthesis loading is an emerging method for making surgical and custom prosthesis design decisions in a more objective manner. Such predictions would necessitate the estimation of forces generated by muscles spanning the lower trunk and all joints of the lower extremities. However, estimating trunk and leg muscle forces simultaneously during walking based on electromyography (EMG) data remains challenging due to the limited number of EMG channels typically used for measurement of leg muscle activity. This study developed a computational method for estimating unmeasured trunk muscle activations during walking using lower extremity muscle synergies. To facilitate the calibration of an EMG-driven model and the estimation of leg muscle activations, EMG data were collected from each leg. Using non-negative matrix factorization, muscle synergies were extracted from activations of leg muscles. On the basis of previous studies, it was hypothesized that the time-varying synergy activations were shared between the trunk and leg muscles. The synergy weights required to reconstruct the trunk muscle activations were determined through optimization. The accuracy of the synergy-based method was dependent on the number of synergies and optimization formulation. With seven synergies and an increased level of activation minimization, the estimated activations of the erector spinae were strongly correlated with their measured activity. This study created a custom full-body model by combining two existing musculoskeletal models. The model was further modified and heavily personalized to represent various aspects of the pelvic sarcoma patient, all of which contributed to the estimation of trunk muscle activations. This proposed method can facilitate the prediction of post-surgery walking function and pelvic prosthesis loading, as well as provide objective evaluations for surgical and prosthesis design decisions.

## Introduction

Pelvic sarcomas account for as much as 10% of osteosarcoma cases and disproportionately affect individuals under the age of 25 (Morris, 2010). Thanks to advances in medical imaging, limb-salvaging internal hemipelvectomy surgery (Lackman et al., 2009) has become common for treating pelvic sarcomas (Lewis, 2014; Puchner et al., 2017). This surgery involves removal of the cancerous tumor on the affected side of the pelvis, which in turn involves removal of the pelvic bone, hip muscles, and trunk muscles infiltrated by the tumor. In many cases, the hip joint is also infiltrated, necessitating removal of the acetabulum and femoral head as well. Planning of internal hemipelvectomy surgery remains challenging due to the highly unique characteristics of each patient’s clinical situation.

When the hip joint must be removed, the orthopedic oncologist typically has two options for surgical restoration of ambulatory function. The first option involves no reconstruction of the hip joint. With this option, the surgeon attaches the proximal femur to remaining pelvic bone using surgical wire. During recovery, the region is immobilized over a period of months so that scar tissue can form, creating a pseudo-hip joint that can bear the patient’s weight and permit ambulation. The advantages of this option are minimization of complications and a low risk of revision surgery, while the disadvantages are abnormal and somewhat limited ambulatory function along with a significant limb length discrepancy that often produces low back pain and scoliosis (Wedemeyer and Kauther, 2011). The second option involves reconstruction of the hip joint using a custom pelvic prosthesis and total hip replacement. With this option, an orthopedic implant company provides a custom pelvic prosthesis that typically recapitulates the removed pelvic bony anatomy, permitting implantation of a total hip replacement that restores hip function. The advantages of this option are a more normal gait pattern with no limb length discrepancy and a shorter recovery time, while the disadvantages are an increased risk of complications and revision surgery due to prosthesis failure (Henshaw and Malawer, 2004; Henderson et al., 2011).

For these two surgical options, the orthopedic oncologist and (when relevant) orthopedic implant company must make numerous decisions related to how the surgery and custom pelvic prosthesis should be designed so as to maximize post-surgery ambulatory function while minimizing the risk of custom prosthesis failure (Guo et al., 2010; Chao et al., 2015). For no reconstruction surgery, decisions include where to attach the proximal femur within the remaining pelvic bone and whether to keep certain muscles (e.g., the psoas) whose retention is surgically challenging and prolongs time in the operating room. For custom prosthesis reconstruction surgery, decisions include how the custom pelvic prosthesis should be designed, where the hip center should be located in the custom prosthesis (the original anatomic location may not be the best choice given that numerous hip and trunk muscles are removed), and where and how screws should be located to attach the custom prosthesis to remaining pelvic bone. Despite significant patient heterogeneity, orthopedic oncologists and orthopedic implant companies currently use subjective experience to make these decisions, which raises the possibility that better functional outcomes could be achieved if these decisions were made using a more objective approach.

An emerging option for making surgical and custom prosthesis design decisions more objectively involves the use of personalized electromyography (EMG)-driven neuromusculoskeletal computer models. Computational modeling and simulation technology has reached the point that personalized neuromusculoskeletal models can be constructed from a patient’s movement (e.g., video motion capture, ground reaction, EMG) and imaging (e.g., CT, MR) data (Valente et al., 2017; Modenese et al., 2018) and used to predict how planned interventions will affect the patient’s post-surgery movement function (Pitto et al., 2019; Sauder et al., 2019; Fregly, 2021). To date, such models have been used to predict the influence of various orthopedic procedures (e.g., derotation osteotomy, muscle transfer, muscle release, patellar advancement) on walking function for individuals with cerebral palsy (Pitto et al., 2019) and the optimal muscle functional electrical stimulation design to maximize paretic leg propulsion for an individual post-stroke (Sauder et al., 2019). To implement a similar approach for individuals with pelvic cancer, researchers must be able to predict post-surgery walking function and custom pelvic prosthesis loading, including artificial hip joint contact force, for different surgical and custom prosthesis designs under consideration. Such predictions would in turn require estimating post-surgery forces generated by muscles spanning the lower trunk and all lower extremity joints. However, predicting trunk and leg muscle forces concurrently during walking based on EMG data remains challenging, since the limited number of experimental EMG channels are typically used to measure the activity of leg muscles, with EMG data rarely being collected from trunk muscles as well during walking (Callaghan et al., 1999; White and McNair, 2002; Anders et al., 2007).

This study develops and performs an initial evaluation of a computational method for estimating unmeasured trunk muscle activations during walking using lower extremity muscle synergies extracted from measured leg muscle EMG data. Muscle synergies are a low-dimensional representation (typically four to six signals) of a larger number of experimentally measured muscle activations (typically eight to 16 signals), where each muscle synergy is composed of a single time-varying synergy activation plus a corresponding time-invariant synergy vector containing weights that define how the synergy activation contributes to the activations of all muscles (D’Avella et al., 2003; Tresch et al., 2006; Tresch and Jarc, 2009). Recent walking studies have demonstrated that five or six synergy activations extracted from 15 leg muscle EMG signals can be linearly combined to predict reliably the activation of another leg muscle with missing EMG data (Ao et al., 2020; Ao et al., 2022). However, this approach has only been applied when EMG data are available from multiple muscles spanning each modeled joint. The present study seeks to extend this computational methodology to a region of the body where no EMG data are typically available based on the observation that leg and trunk muscles appear to share common synergy activations (Ivanenko et al., 2004; Torres-Oviedo and Ting, 2007; Saito et al., 2021). The methodology was developed using 32 channels of EMG data (16 channels per side) collected during walking from a single pelvic sarcoma patient prior to surgery. Fifteen channels per side were collected from leg muscles and used to calculate synergy activations, while the remaining channel per side was collected from a single trunk muscle (erector spinae) and used to evaluate the proposed computational methodology.

## Methods

### Experimental data collection

Experimental walking data were collected prior to surgery from a single subject (sex: male, age: 46 years, height: 1.73 m, mass: 85 kg) diagnosed with a pelvic sarcoma in the pubic and acetabular regions of the right hemipelvis. The protocol for data collection was approved by the institutional review boards of the University of Texas Health Science Center and MD Anderson Cancer Center, and the subject provided written informed consent. The subject completed a static standing trial with feet pointing forward, followed by a 2-min walking trial on a split-belt instrumented treadmill with belts tied at their self-selected speed of 1 m/s. Collected experimental data included video motion capture (Qualisys AB, Gothenburg, Sweden), ground reaction (Bertec Corp., Columbus, OH, United States), and wireless electromyography (EMG) (Cometa, Bareggio, Italy). Twelve static markers were placed to approximate toe axis, ankle axis, and knee axis of each leg for model scaling purposes, while 36 dynamic markers (5 on the torso, 2 on each arm, 3 on the pelvis, 3 on each thigh, 1 on each patella, 4 on each shank, and 4 on each foot) were placed to define the position and orientation of each body segment during gait motion. EMG data were collected from the muscles in each leg using 15 channels (Table 1), and from the erector spinae muscle group using another channel on each side. We were unable to collect the EMG signal of the right iliopsoas muscle using a fine-wire electrode due to its proximity to the tumor tissues. Data from ten gait cycles were selected for analysis, with each cycle defined by two consecutive right foot strikes. The subject’s CT imaging data for the pelvic region was also made available.

**TABLE 1:** List of lower extremity muscles in the musculoskeletal model, the muscle excitation signal sources, and the method used to acquire the muscle excitation signals.

Muscle	Muscle excitation signal source	Muscle excitation acquisition method
Adductor brevis	Adductor longus	Measured EMG
Adductor longus		
Adductor magnus distal		
Adductor magnus ischial		
Adductor magnus middle		
Adductor magnus proximal		
Gracilis		
Biceps femoris long head	Bicep femoris long head	Measured EMG
Biceps femoris short head		
Gemelli	Quadratus femoris	Synergy Extrapolation
Quadratus femoris		
Gluteus maximus superior	Gluteus maximus	Measured EMG
Gluteus maximus middle		
Gluteus maximus inferior		
Piriformis		
Gluteus medius anterior	Gluteus medius	Measured EMG
Gluteus medius middle		
Gluteus medius posterior		
Gluteus minimus anterior		
Gluteus minimus middle		
Gluteus minimus posterior		
Iliacus	Iliopsoas	Measured EMG
Psoas major superior		Synergy Extrapolation^a^
Psoas major middle		
Psoas major inferior		
Rectus femoris	Rectus femoris	Measured EMG
Sartorius	Sartorius	Synergy Extrapolation
Pectineus		
Semimembranosus	Semimembranosus	Measured EMG
Semitendinosus		
Tensor fasciae latae	Tensor fasciae latae	Synergy Extrapolation
Vastus medialis	Vastus medialis	Measured EMG
Vastus intermedius		
Vastus lateralis	Vastus lateralis	Measured EMG
Extensor digitorum longus	Extensor digitorum longus	Synergy Extrapolation
Extensor hallucis longus		
Flexor digitorum longus	Flexor digitorum longus	Synergy Extrapolation
Flexor hallucis longus		
Gastrocnemius lateral	Gastrocnemius lateral	Measured EMG
Gastrocnemius medial	Gastrocnemius medial	Measured EMG
Peroneus brevis	Peroneus longus	Measured EMG
Peroneus longus		
Soleus	Soleus	Measured EMG
Tibialis anterior	Tibialis anterior	Measured EMG
Tibialis posterior	Tibialis posterior	Measured EMG

^a^ Fine wire EMG, data were available from iliopsoas for only the left leg. data from iliopsoas for the right leg was not possible due to proximity to the cancerous tumor. The muscle excitations of the right iliopsoas muscle were estimated using synergy extrapolation instead.

### Generic musculoskeletal model development

**FIGURE 1:**
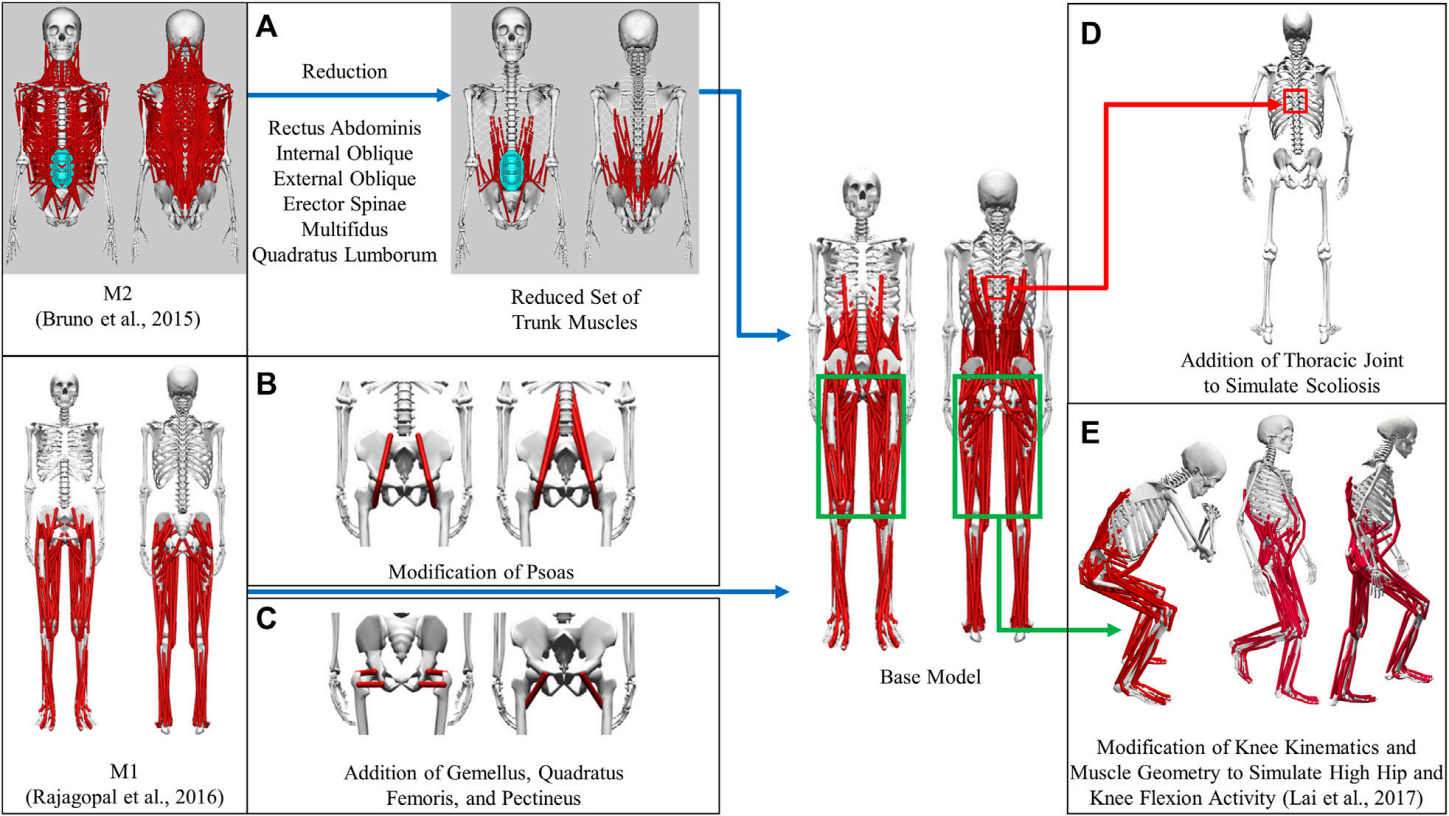
Development of the base model from generic models. **(A)** Transfer of a reduced set of trunk muscles from M2 to the base model. **(B)** Modification of psoas to place muscle origins on the spine and splitting the single-head psoas in M1 into the eventual 3 heads **(C)** Addition of gemellus, quadratus femoris, and pectineus to the base model **(D)** Creation of the thoracic joint to simulate scoliosis **(E)** Modification of knee joint kinematics and muscles to simulate high flexion activities.

A full-body musculoskeletal model (henceforth named the base model, Figure 1) was obtained by combining two commonly used generic models in OpenSim (Delp et al., 2007; Seth et al., 2018). The first model (M1) is predominantly used for simulating gait (Rajagopal et al., 2016) and the second model (M2) for thoracolumbar spine and rib cage movement (Bruno et al., 2015). The base model was capable of independently simulating trunk muscle force generation during gait. The combination process could be summarized as mainly the transfer of the trunk muscles from M2 into M1. There were, however, additional modifications made to the base model in order to make it more suitable for our simulation requirements; these modifications are detailed below. As a result of these modifications, a full-body musculoskeletal model capable of simulating the force generation of trunk muscles was developed. This model consisted of 148 rigid tendon Hill-type MTUs (29 on each side of the trunk and 45 in each leg), which actuated movement in the spine and lower extremity joint degrees of freedom (DoFs): thoracic (3 DoFs), lumbosacral (3 DoFs), and each hip (3 DoFs), knee (2 DoFs), ankle (1 DoF), and subtalar (1 DoF).

#### Base model muscle modifications

The muscles spanning the lumbosacral joint in M2 were identified as the trunk muscles needed to be added into the base model (Figure 1A). The trunk muscles included rectus abdominis (RA), external oblique (EO), internal oblique (IO), erector spinae (ES), multifidus (MF), and quadratus lumborum (QL). The original set of 71 Hill-type muscle-tendon units (MTUs) per side in M2 (Table 2) was identified for transferal. The transferal of muscle geometry between the models was completed using nmsBuilder (Valente et al., 2017) and following a codified workflow (Modenese et al., 2018). The workflow included the following steps: First, the same set of bony landmarks on the spine, ribcage and pelvis was identified in both M1 and M2. Next, the affine transformation used to project the landmarks in M2 onto those in M1 was determined and then applied to map the muscle path points from M2 onto the base model. Lastly, adjustments were made to ensure that the transferred muscle path points in the base model were positioned accurately. According to previous research, it was likely that the large number of MTUs in the original set would complicate any model-based motion prediction as one of the main reasons of this model customization effort (Meyer et al., 2016; Sauder et al., 2019). Therefore, an optimization-based model reduction method (Vega et al., 2022) identified the reduced set of MTUs with modified peak isometric force that, for a large number of random poses, would generate joint moments highly comparable to those generated by the original set. The reduced trunk muscle group would have only 29 MTUs per side (Table 2).

**TABLE 2:** Original set of trunk muscles included in the published OpenSim lumbar spine model (Bruno et al., 2015) and the reduced set included in the modified OpenSim full-body model. ACSA is anatomical cross-section area in cm^2^, FMo is peak isometric force in N, lMo is optimal muscle fiber length in cm, and lTs is tendon slack length in cm. G indicates a linearly scaled generic value, and P indicates a personalized value. Muscles listed in the first column are rectus abdominis (RA), erector spinae (ES), quadratus lumborum (QL), multifidus (MF), external oblique (EO), and internal oblique (IO).

	Original lumbar spine model			Reduced full-body model							
		ACSA			ACSA		FMo	lMo		lTs	
		G	P		G	P	P	G	P	G	P
RA	Original Set			Reduced Set							
	rect_abd	6.62	7.40	rect_abd	6.62	7.50	750	34.11	34.99	9.28	9.33
ES	Original Set			Reduced Set							
	IL_L1	1.47	1.68								
	IL_L2	1.83	2.53	IL_L2	2.95	3.94	394	4.06	3.69	9.20	8.22
	IL_L3	2.17	3.10								
	IL_L4	4.15	5.41	IL_L4	6.68	8.92	892	2.01	1.74	4.55	3.35
	IL_R5	0.57	0.30								
	IL_R6	0.73	0.40								
	IL_R7	0.88	0.54	IL_R7	2.73	2.57	257	17.89	17.16	18.80	18.01
	IL_R8	0.78	0.49								
	IL_R9	0.96	0.78								
	IL_R10	1.92	1.68	IL_R10	3.34	3.16	316	15.58	14.52	9.17	8.57
	IL_R11	2.35	2.78	IL_R11	2.93	2.77	277	13.66	11.90	6.84	6.11
	IL_R12	2.06	2.63	IL_R12	1.25	1.18	118	10.33	8.77	5.17	4.48
	LTpT_T7	0.80	0.42								
	LTpT_T8	1.20	0.76	LTpT_T8	2.94	2.23	223	14.27	13.66	22.54	21.53
	LTpT_T9	1.39	0.91								
	LTpT_T10	1.39	0.96								
	LTpT_T11	1.15	1.02								
	LTpT_T12	0.94	1.00	LTpT_T12	3.76	2.85	285	8.78	7.83	14.88	13.04
	LTpT_R7	0.80	0.44								
	LTpT_R8	1.30	0.73	LTpT_R8	3.06	2.39	239	12.21	11.83	24.91	24.28
	LTpT_R9	1.11	0.63								
	LTpT_R10	1.20	0.95								
	LTpT_R11	1.15	1.22	LTpT_R11	3.45	2.69	269	11.55	10.03	16.45	14.04
	LTpT_R12	0.94	1.07								
	LTpL_L1	1.06	1.35								
	LTpL_L2	1.08	1.50	LTpL_L2	1.67	2.55	255	7.62	6.72	8.38	7.32
	LTpL_L3	1.21	1.73								
	LTpL_L4	1.52	1.98	LTpL_L4	2.34	3.57	357	4.70	3.89	5.06	4.14
	LTpL_L5	1.58	3.14	LTpL_L5	2.44	3.71	371	2.54	4.06	0.05	0.10
QL	Original Set			Reduced Set							
	QL_post_I_1-L3	0.76	0.99	QL_post_I1_L3	0.76	1.00	100	3.74	3.85	4.45	3.23
	QL_post_I_2-L2	0.37	0.28								
	QL_post_I_2-L3	0.59	0.77								
	QL_post_I_2-L4	1.56	2.79	QL_post_I2_L4	2.51	3.87	387	2.38	2.58	2.84	2.10
	QL_post_I_3-L1	0.77	0.56								
	QL_post_I_3-L2	0.56	0.44	QL_post_I3_L2	2.30	1.81	181	4.86	5.22	5.78	4.37
	QL_post_I_3-L3	0.96	0.81								
	QL_ant_I_2-T12	0.45	0.22	QL_ant_I2_T12	0.73	0.57	57	5.81	10.08	11.18	5.33
	QL_ant_I_2-12_1	0.28	0.35								
	QL_ant_I_3-T12	0.85	0.66								
	QL_ant_I_3-12_1	0.53	0.41								
	QL_ant_I_3-12_2	0.35	0.27	QL_ant_I3_R12_I2	2.14	1.68	168	5.07	8.57	9.76	4.46
	QL_ant_I_3-12_3	0.41	0.32								
MF	Original Set			Reduced Set							
	MF_m1t_2	0.60	0.54								
	MF_m1t_3	1.00	0.92	MF_m1t_3	1.59	1.47	147	12.00	9.85	3.60	3.15
	MF_m2s	0.54	0.44								
	MF_m2t_1	0.57	0.47								
	MF_m2t_2	1.46	1.23								
	MF_m2t_3	1.61	1.36	MF_m2t_3	4.19	3.52	352	10.69	8.60	3.25	2.82
	MF_m3s	0.84	0.70	MF_m3s	3.57	3.10	310	6.39	4.52	2.66	1.95
	MF_m3t_1	0.91	0.79								
	MF_m3t_2	0.91	0.79								
	MF_m3t_3	0.91	0.79								
	MF_m4s	1.01	0.92								
	MF_m4t_1	0.90	0.82								
	MF_m4t_2	0.90	0.82								
	MF_m4t_3	0.90	0.82	MF_m4t_3	3.97	3.62	362	9.40	7.34	3.81	3.19
	MF_m4_laminar	0.26	0.24								
	MF_m5s	0.35	0.35								
	MF_m5t_1	0.35	0.35								
	MF_m5t_2	0.35	0.35								
	MF_m5t_3	0.35	0.35								
	MF_m5_laminar	0.56	0.56	MF_m5_laminar	1.95	1.95	195	3.94	2.53	1.50	0.99
EO	Original Set			Reduced Set							
	E0_R10	1.41	1.52	EO10	2.09	2.30	230	10.46	10.47	1.16	1.06
	E0_R11	1.41	1.53								
	E0_R12	1.53	1.66	EO12	2.26	2.49	249	9.26	8.72	1.03	0.88
IO	Original Set			Reduced Set							
	IO1	1.96	3.10								
	IO2	2.02	2.62	IO2	5.90	7.82	782	11.63	11.92	1.29	1.28
	IO3	1.92	1.92								
	IO4	2.33	2.33	IO4	3.29	3.34	334	11.15	11.35	1.24	1.22
	IO5	2.04	2.04	IO5	2.89	2.93	293	8.01	8.19	0.89	0.88
	IO6	1.80	1.80								

The MTUs representing psoas major muscle group in the base model were a combination of the psoas muscle representations (Figure 1B) in M1 and M2. The single-head psoas muscle in M1 was split into the 11 heads in M2 with their origins on the spine between L1 and L5. The representation of psoas wrapping around the anterior part of the pelvic brim and ending with insertion on the lesser trochanter of the femur in M1 (geometric path points 2, 3, 4 and wrapping object PS_at_brim) was maintained and replicated for each of the 11 psoas muscle heads. The volumetric fraction ratio of the 11 muscle heads in M2 was used to divide the total psoas muscle volume in M1 in order to determine the peak isometric force of each head. Finally, using the aforementioned model reduction procedure, we reduced the number of psoas muscle heads in the initial model to three.

Gemellus, pectineus, and quadratus femoris were added to the base model (Figure 1C). The origin-to-insertion paths, tendon slack lengths, and optimal fiber lengths for these muscles were extracted directly from a model preceding M1 (Arnold et al., 2010) because the musculoskeletal geometry of the two models was highly similar. The incorporation of these muscles could enhance the model’s ability to match inverse dynamic joint moments and provide a more comprehensive set of loading conditions for the finite elemental analysis used in the design of a custom prosthesis implant.

#### Base model joint modifications

We introduced new and modified existing joint definitions in the base model to account for clinical observations and simulation requirements. Resection of the acetabulum and hip abductor muscles on the operated side is the most common surgical procedure for pelvic sarcoma patients at the MD Anderson Cancer Center. To compensate for the loss of abductors, the patient would likely develop scoliosis at the T8-T9 level following surgery. Therefore, a thoracic joint with three rotational DoFs was added between T8 and T9 vertebrae in the model to simulate the probable scenario of scoliosis (Figure 1D).

The definition of the knee varus-valgus angle was also modified. Previously, the angle of varus-valgus of the knee was modeled as a function of the angle of knee flexion (Walker et al., 1985). To represent varus-valgus at each knee, the knee adduction angle was introduced as a new degree of freedom. The knee adduction angle was no longer dependent on the knee flexion angle and could be adjusted based on the subject’s knee anatomy.

To simulate daily activities involving high flexion of the hip and knee joints, such as squatting and ascending and descending stairs, the original model was modified further (Figure 1E). Knee joint kinematics, knee muscle origin-to-insertion paths, tendon slack lengths, and optimal fiber lengths were updated based on a model refined atop M1 to make it more suitable for simulating high hip and knee flexion activities (Lai et al., 2017).

### Musculoskeletal model personalization

#### Personalization of pelvis geometry

**FIGURE 2:**
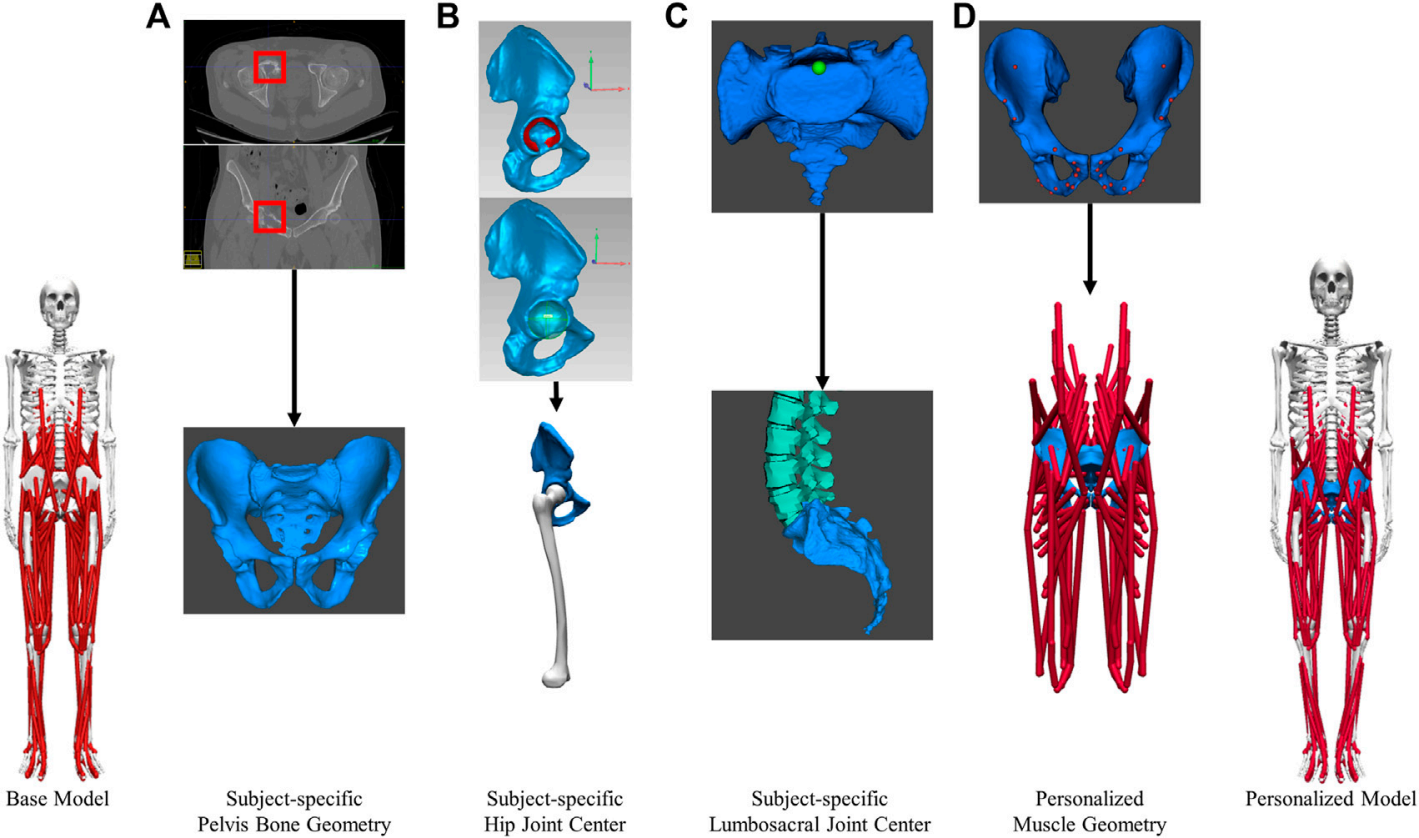
Image-based modeling approach to create subject-specific musculoskeletal model for the pelvic region. **(A)** segmenting CT scan images to obtain pelvis bone geometry. Tumor growth was highlighted by the red boxes **(B)** Determining the hip joint center **(C)** Determining the lumbosacral joint center. **(D)** Creating personalized muscle geometry.

A subject-specific musculoskeletal geometry was obtained by using image-based modeling (Figure 2A). CT scan images were segmented using ITK-SNAP to construct the pelvis geometry (Yushkevich et al., 2006). It was then repositioned and reoriented such that its coordinate system aligned with the one in M1. The articular surface of the hip joint on each side was carefully selected for the sphere fitting tool in Geomagic (3D Systems, Morrisville, NC, United States) to determine the hip joint center location (Figure 2B). The location of lumbosacral joint was identified as the posterior point of the articular surface of the joint (Figure 2C).

The base model was scaled to match the subject anthropometrically using the OpenSim Scale Model tool. With the exception of the pelvis, the dimensions of various body segments were scaled based on the marker data collected during the static standing trial. The pelvis was manually scaled using pre-calculated scale factors to ensure that the hip and back joint centers in the scaled model coincide with those found in the subject-specific pelvis geometry previously. The average varus-valgus angle for each knee during the static standing trial was set as the default knee adduction angle and locked during subsequent analyses.

The musculoskeletal geometry of the pelvis in the scaled base model was replaced by the subject-specific geometry. Firstly, the right and left hemipelvis geometries scaled from generic geometry were replaced by the corresponding image-based geometries. The generic sacrum bone geometry was retained and aligned with the image-based sacrum using the Geomagic global registration tool. The resulting sacrum bone geometry closely matched the image-based geometry in shape and maintained a comparable sacroiliac joint angle as the subject. Secondly, after the replacement of bone geometry, the muscle attachments and path points defined in the proximity of the right or left hemipelvis were updated (Figure 2D) using the previously described muscle transferal workflow (*Base Model Muscle Modifications Section*). Bony landmarks on the generic hemipelvis geometry were mapped to those on the image-based geometry to determine the affine transformation. Separately, the muscle attachments and path points defined in close proximity to the sacrum, were updated by applying the transformation used to register the generic sacrum geometry onto the image-based geometry. All the muscle attachments were carefully adjusted so that they remained on the surface of the subject-specific pelvis and sacrum bone geometry.

#### Personalization of lower extremity muscles

An enhanced EMG-driven model calibrated the muscle-tendon model parameters for each muscle in the lower extremities of the geometry-personalized musculoskeletal model (Table 1), where the experimentally unmeasured muscle excitations were predicted by “synergy extrapolation” (Meyer et al., 2017; Ao et al., 2020; Ao et al., 2022). The EMG-driven model took inputs of both measured and unmeasured muscle excitations, residual muscle excitations, and joint kinematics from OpenSim inverse kinematics analysis, where unmeasured muscle excitations and residual muscle excitations (being only applied to measured muscle excitations), were constructed by linearly combining unknown synergy vector weights and time-varying synergy excitations extracted from measured muscle excitations. A multi-objective optimization simultaneously calibrated the activation dynamics model parameters (EMG scale factor, electromechanical delay, activation time constant, and activation non-linear shape factor), the Hill-type muscle-tendon model parameters (optimal muscle fiber length and tendon slack length) for each muscle-tendon unit in the lower-extremity model, and synergy vector weights associated with unmeasured and residual muscle excitations such that the EMG-driven joint moments at 6 low-extremity DoFs (3 for hip, 1 for knee and 2 for ankle joints) would primarily match the OpenSim inverse dynamics joint moments as closely as possible. The optimization problem was formulated as a trade-off between prediction accuracy of joint moments and magnitude minimization of unmeasured and residual muscle excitations. Prediction of residual muscle excitations applied to measured muscle excitations was included during optimization to improve the predictive accuracy of unmeasured muscle excitations. After the EMG-driven model was calibrated, it was used to estimate the activations and forces of all the muscles in the lower extremities.

#### Personalization of trunk muscles

Three Hill-type muscle tendon model parameters for the 58 trunk muscles in the model were personalized. Firstly, the peak isometric force of each trunk muscle was personalized using published regression models (Anderson et al., 2012). Using inputs of the subject’s gender, age, height and mass, the anatomical cross-sectional areas (ACSAs) of the subject’s trunk muscle groups at vertebral levels between T6 and L5 were estimated by the regression models. Individual MTU’s ACSA was calculated using the method used to develop subject-specific models from M2 (Bruno et al., 2017). The total ACSA for each trunk muscle group was distributed to the MTUs representing the muscle group in the reduced set based on the ACSA fraction ratio between the MTUs in the reduced set. The ACSA for each trunk MTU in the reduced set was multiplied by the maximum muscle stress of 100 N/cm^2^ (Bruno et al., 2015) to obtain the peak isometric force (Table 2). Secondly, the tendon slack length and optimal fiber length of each trunk MTU were adjusted (Table 2) so that the normalized length of the MTU during gait was slightly less than 1 for close-to-optimal force generation (Supplementary Figure S1).

### Trunk muscle activation estimation

#### Lower extremity muscle synergy analysis

The lower extremity muscle activations were decomposed to find a lower-dimensional set of time-varying synergy activations and corresponding time-invariant synergy vectors (Meyer et al., 2016). The algorithm used for the synergy extraction was non-negative matrix factorization (NMF), which was implemented using MATLAB “nnmf” function (MathWorks, Natick, MA). For each of the 10 selected gait cycles, the lower extremity muscle activations in each leg were decomposed into 5, 6, 7, and 8 synergies because there was evidence that 5 muscle synergies were sufficient during gait (Ivanenko et al., 2004). Our goal was to investigate whether increasing the number of synergies could affect the estimation of trunk muscle activations. Each synergy activation was normalized by its maximum such that each normalized synergy activation had a maximum value of 1.

#### Optimization formulation

**FIGURE 3:**
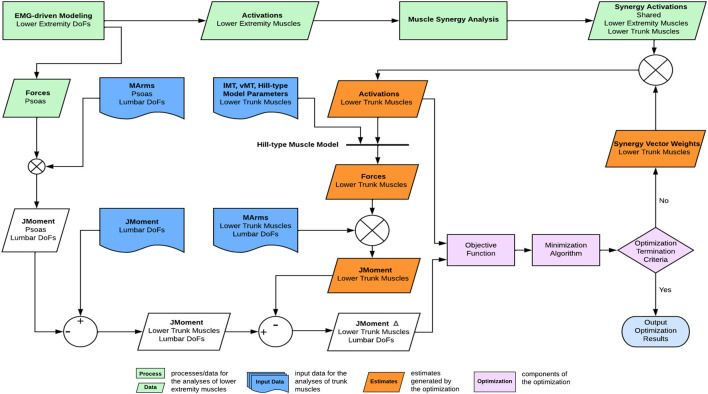
Overview of the computational framework used to estimate trunk muscle activations and forces. From lower extremity muscle activations, muscle synergies were extracted and of which, the synergy activations were then combined with synergy vector weights of the trunk muscles that were searched iteratively by the optimization until the optimal solution of trunk muscle activations was found.

A nonlinear optimization problem estimated the trunk muscle activations constructed from lower extremity synergy activations such that the three resulting lumbosacral joint moments closely matched those calculated from inverse dynamics (Figure 3). The synergy activations extracted from ipsilateral lower extremity muscles were assumed to be shared by ipsilateral trunk muscles (Ivanenko et al., 2004; Saito et al., 2021). The only remaining unknowns for estimating the trunk muscle activations were the synergy vector weights for the trunk muscles. As a result, the synergy vector weights for the trunk muscles were iteratively adjusted within the optimization until the optimal solution for the trunk muscle activations was found.

The optimization problem described below searched for optimal trunk muscle activations, which was carried out using the MATLAB “fmincon” function.

$$\min_{w_{est}}J=J_{M}+J_{a}+J_{a\;dev}+J_{w\;dev}$$

subject to bounds $w_{est}\geq 0$ and linear inequality constraints $0\leq a_{est}\leq 1$

Where $w_{est}$ is the vector of design variables, the synergy vector weights of the trunk muscles, and $a_{est}$ is the vector of estimated trunk muscle activations. Expanding each of the terms in the objective function,

$$J_{M}=\frac{1}{nPt∙nDoF}\overset{nPt}{\underset{i=1}{\sum }}\overset{nDoF}{\underset{j=1}{\sum }}{\left(\frac{{M_{est}}_{i,j}-{M_{ID}}_{i,j}}{Allow_{∆M}}\right)}^{2}$$

where, $M_{est}$ is the model-estimated lumbosacral joint torques produced by the 58 trunk MTUs, $M_{ID}$ is the inverse dynamic lumbosacral joint torques excluding contributions by the psoas, nPt is the number of time instants analyzed, and nDoF is the number of DoFs. This cost term penalizes the difference between the estimated and inverse dynamic lumbosacral joint moments, especially when the difference is greater than the acceptable value $Allow_{∆M}$ .

$$J_{a}=\frac{1}{nPt∙nMusc}\overset{nPt}{\underset{i=1}{\sum }}\overset{nMusc}{\underset{j=1}{\sum }}{\left(\frac{a_{est_{i,j}}}{Allow_{a}}\right)}^{2}$$

where $a_{est}$ is the model-estimated muscle trunk activations, and nMusc is the number of trunk muscles. This term seeks to minimize the estimated trunk muscle activation. The cost associated with this term becomes more severe when $a_{est}$ is above the activation threshold $Allow_{a}$ .

$$J_{a\;dev}=\overset{nGrp}{\underset{i=1}{\sum }}\left[\frac{1}{nPts∙nMiGrp}\overset{nPts}{\underset{j=1}{\sum }}\overset{nMiGrp}{\underset{k=1}{\sum }}{\left(\frac{{a_{est}}_{j,\left(i,k\right)}-{\bar{a}}_{estGrp_{j,i}}}{Allow_{∆a}}\right)}^{2}\right]$$

where the trunk MTUs were grouped into 6 large muscles (RA, EO, IO, ES, MF, and QL, see Table 2 ${a_{est}}_{j,\left(i,k\right)}$ is the activation of the *k^th^* head of the *i^th^* large muscle, and ${\bar{a}}_{estGrp_{j,i}}$ is the mean activation across the heads of the *i^th^* muscle, both computed at the *j^th^* time instant, nMiGrp is the number of heads for the muscle. This term penalizes the difference between the activations of individual muscle head and the mean across the muscle heads. Penalties become especially steep when the differences are larger than the allowable value $Allow_{∆a}$ .

The allowable value for each cost term was determined using the following logic. $Allow_{∆M}$ was set to 5 Nm comparable to the errors found in the estimated lower extremity joint moments (Meyer et al., 2017; Ao et al., 2020; Ao et al., 2022). $Allow_{a}$ was set to 1.00, 0.75, 0.50, and 0.25 to create four cases to investigate how muscle activation cost term could affect the performance of estimation. The $Allow_{∆a}$ and $Allow_{∆w}$ were both set to 0.05 so that the various heads of the same muscle have comparable activations and synergy structures.

#### Evaluation

Two metrics were used to assess the activation and force estimates of the trunk muscles. First, the root mean squared error (RMSE) between the lumbosacral joint moments generated by the estimated trunk muscle forces and those from inverse dynamics (excluded psoas contribution) was calculated. The second comparison was between the estimated muscle activations of ES and its measured and processed muscle excitations. The second metric was determined by calculating the Pearson correlation coefficient *r* between the average curves of the estimated ES activations and the electromechanically delayed ES excitations. Electromechanical delay (EMD) was implemented to bridge the temporal gap between muscle excitations and activations. EMD values for ES were found to be affected by the rate of force generation and electrode placement, with a mean and standard deviation of 133.1 and 29.8 ms at L1 and 135.1 and 32.8 ms at L2 (van Dieën et al., 1991). To account for the reported range of EMD values and the uncertainty in electrode placement during experimental data collection, a range of EMDs from 100 to 165 ms with a 5 ms increment was applied to the measured muscle excitations of ES, and the EMD value of ES was allowed to vary between sides. A post-analysis evaluation would identify the highest correlation between the estimated activations and the electromechanically delayed muscle excitations.

The trunk muscle activations and forces were also estimated using a commonly formulated static optimization that minimized the sum of the squares of muscle activations (Anderson and Pandy, 2001; Shourijeh and Fregly, 2020). For more information on the formulation, please see the Supplementary Material. The static optimization results were compared to our estimates.

## Results

**FIGURE 4:**
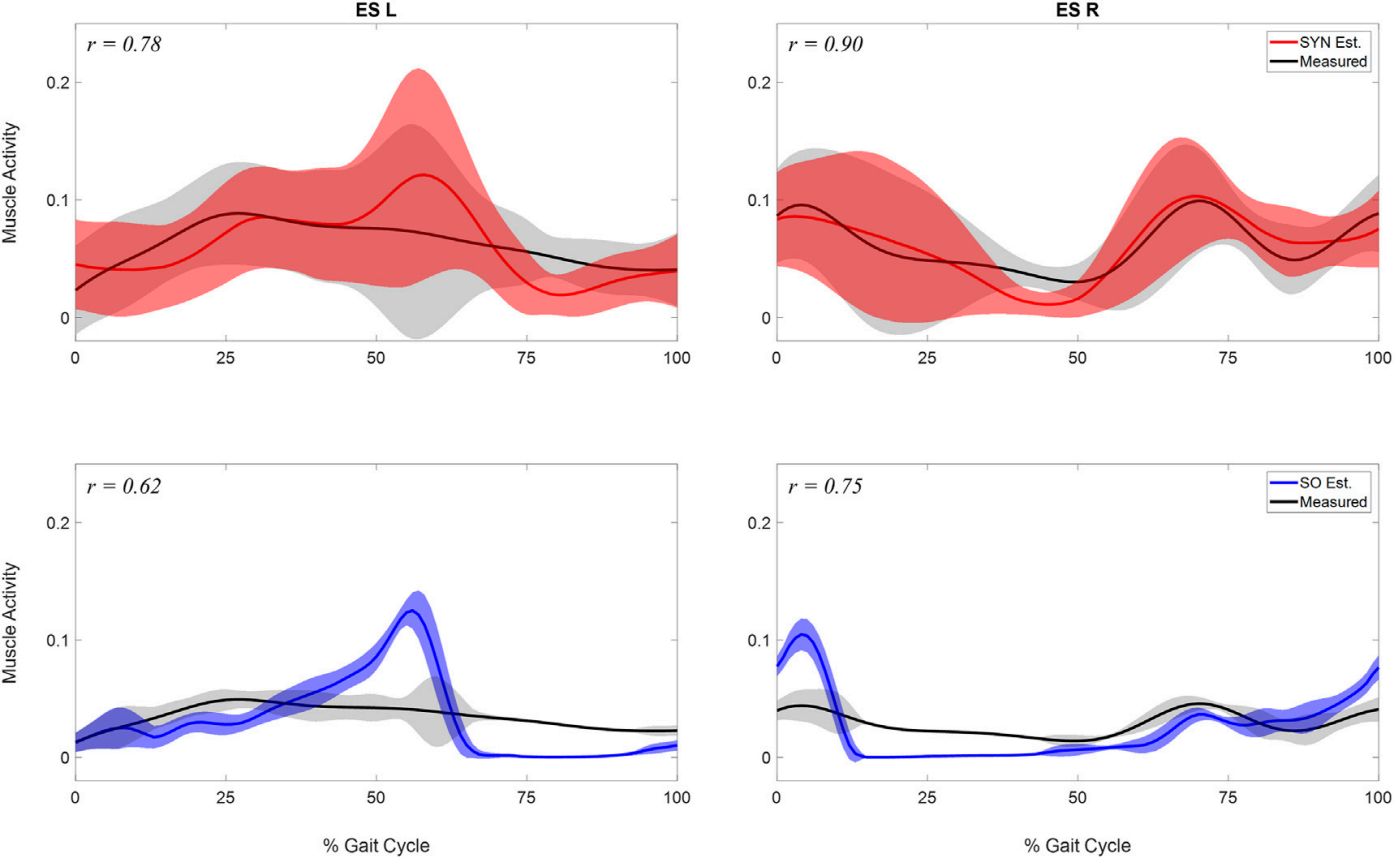
Comparison of estimated and measured muscle activity of erector spinae left (ES L) and right (ESR). Mean and standard deviation of muscle activations across the 10 analyzed gait cycles were represented by the curve and the shaded area respectively. The activations were estimated by two methods: 1. the synergy-based method proposed in study (SYN Est., shown in the first row), using 7 synergies and $Allow_{a}=0.50$ ; 2. the static optimization method (SO Est., shown in the second row). For the purpose of display and comparison, the mean of standard deviation of measured muscle excitations (represented by the black curve and grey shaded area) were both electromechanically delayed and scaled to minimize the difference with the estimated activations. The correlations between the mean curves of estimated and measured muscle activity were displayed at the top left corner.

When the number of synergies was 7, the estimated activations of the erector spinae right (ES R) and left (ES L) correlated strongly (*r* > 0.7 (Moore et al., 2015)) with the measured muscle excitations (Table 3). Moreover, when $Allow_{a}$ was set to 0.50 in the optimization, the correlation between the mean curves of the estimated and the measured activations for ES R was the highest (*r* = 0.90), and the correlation for ES L was also strong (*r* = 0.78) (Table 3; Figure 4). When the number of synergies increased from 7 to 8, and $Allow_{a}$ remained at 0.5, the correlation for both ES R and ES L decreased. In the case of static optimization (SO), the correlation between estimated and measured muscle activity was moderate (*r* = 0.62) for ES L and strong (*r* = 0.75) for ES R (Table 3; Figure 4).

**TABLE 3:** The Pearson correlation coefficient, ***r***, between the mean curves of the estimated activations and the measured muscle excitations of the erector spinae of the left side (ES L) and the right side (ES R) across the 10 gait cycles selected for analysis. Two estimation methods were compared, the proposed synergy-based approach (SYN) using 5–8 muscle synergies per side and static optimization (SO). $Allow_{a}$ indicated the allowable activation value used in the optimization formulation to minimize activation (*Optimization Formulation Section*).

Method	Number of synergies		*r*	
			ES L	ES R
SYN	5	$Allow_{a}=1.00$	0.69	0.59
		$Allow_{a}=0.75$	0.66	0.62
		$Allow_{a}=0.50$	0.64	0.66
		$Allow_{a}=0.25$	0.54	0.78
	6	$Allow_{a}=1.00$	0.44	0.59
		$Allow_{a}=0.75$	0.50	0.59
		$Allow_{a}=0.50$	0.64	0.63
		$Allow_{a}=0.25$	0.56	0.75
	7	$Allow_{a}=1.00$	0.77	0.74
		$Allow_{a}=0.75$	0.79	0.80
		$Allow_{a}=0.50$	0.78	0.90
		$Allow_{a}=0.25$	0.71	0.87
	8	$Allow_{a}=1.00$	0.82	0.36
		$Allow_{a}=0.75$	0.77	0.45
		$Allow_{a}=0.50$	0.72	0.63
		$Allow_{a}=0.25$	0.64	0.81
SO		$Allow_{a}=1.00$	0.62	0.75

Both the number of synergies and the $Allow_{a}$ value used in the optimization affected the mean RMSE values between the experimental and model-estimated lumbosacral joints (Table 4). The mean RMSE values for a fixed $Allow_{a}$ decreased as the number of synergies increased from 5 to 8. When the number of synergies remained constant, a smaller $Allow_{a}$ increased the mean RMSE values for the estimated moments in lumbosacral extension-flexion and lateral bending but decreased that for lumbosacral rotation.

**TABLE 4:** The average and standard deviation of the root-mean-square error (RMSE) in the lumbosacral joint moments estimated using 5–8 muscle synergies per side across the 10 gait cycles selected for analysis. $Allow_{a}$ indicated the allowable activation value used in the optimization formulation to minimize activation (*Optimization Formulation Section*).

Number of synergies		RMSE (Nm)		
		Lumbar extension	Lumbar bending	Lumber rotation
5	$Allow_{a}=1.00$	4.04 ± 1.03	5.22 ± 0.90	2.46 ± 0.62
	$Allow_{a}=0.75$	4.05 ± 1.02	5.24 ± 0.89	2.45 ± 0.60
	$Allow_{a}=0.50$	4.12 ± 0.99	5.31 ± 0.88	2.42 ± 0.56
	$Allow_{a}=0.25$	4.28 ± 0.97	5.64 ± 0.86	2.40 ± 0.47
6	$Allow_{a}=1.00$	3.22 ± 0.50	4.36 ± 0.62	2.00 ± 0.61
	$Allow_{a}=0.75$	3.30 ± 0.53	4.40 ± 0.60	1.97 ± 0.61
	$Allow_{a}=0.50$	3.40 ± 0.59	4.51 ± 0.58	1.94 ± 0.61
	$Allow_{a}=0.25$	3.66 ± 0.66	4.80 ± 0.60	1.96 ± 0.53
7	$Allow_{a}=1.00$	2.87 ± 0.33	3.45 ± 0.71	2.19 ± 1.48
	$Allow_{a}=0.75$	2.90 ± 0.32	3.51 ± 0.70	2.12 ± 1.31
	$Allow_{a}=0.50$	3.00 ± 0.35	3.67 ± 0.68	2.04 ± 1.07
	$Allow_{a}=0.25$	3.29 ± 0.46	4.05 ± 0.74	1.91 ± 0.60
8	$Allow_{a}=1.00$	2.14 ± 0.41	2.66 ± 0.56	2.30 ± 1.46
	$Allow_{a}=0.75$	2.21 ± 0.41	2.77 ± 0.54	2.20 ± 1.27
	$Allow_{a}=0.50$	2.36 ± 0.41	2.97 ± 0.54	2.05 ± 0.98
	$Allow_{a}=0.25$	2.70 ± 0.45	3.43 ± 0.58	1.83 ± 0.57

Examination of the synergy vector weights of all gait cycles using 7 synergies with $Allow_{a}$ value of 0.50 revealed that trunk muscles minimally recruited at least one synergy (vector weights <0.01) on each side of the body (synergy activations marked in Supplementary Figures S4A,B). This characteristic is evident in synergies 1 and 7 on the right side, as well as synergy 6 on the left, during the 10^th^ gait cycle (Supplementary Figures S5A,B).

## Discussion

This study proposed a novel computational method, named SYN, for estimating trunk muscle activations during gait and conducted an initial evaluation using pre-operative data collected from a patient with pelvic sarcoma. A full-body subject-specific musculoskeletal model was developed based on the patient’s pre-operative data to facilitate the computation method. A calibrated EMG-driven lower extremity musculoskeletal model was used to estimate the activations of the lower extremity muscles, from which synergy activations were extracted. SYN estimated trunk muscle activations by multiplying synergy activations extracted from the ipsilateral leg muscles by the synergy vectors found through optimization. It was found that using 5 to 8 muscle synergies extracted from the muscle activations in each leg could reasonably estimate the activations of ipsilateral trunk muscles. Increasing the number of lower extremity muscle synergies extracted from each leg decreased the RMSE between experimental and model-estimated joint moments. When seven muscle synergies were made available for recruitment to the ipsilateral trunk muscles, a strong correlation was found between estimated and measured muscle activity of the erector spinae. Despite the presence of 7 synergies, at least one muscle synergy on each side was minimally recruited by the trunk muscles in the 10 gait cycles we analyzed.

The estimation accuracy of the ES activations varied based on the number of muscle synergies and the $Allow_{a}$ value used in the optimization formulation. Reconstruction with 7 synergies outperformed the other numbers of synergies evaluated as the measured and estimated activity for both ES R and ES L were strongly correlated for all $Allow_{a}$ values in the optimization (Table 3), whereas reconstruction with 5 or 6 synergies produced mostly moderate correlation, and reconstruction with 8 synergies could only achieve strong correlation for ES on one side. For each number of synergies evaluated, the effect of varying $Allow_{a}$ on the estimation performance was not consistent. At 7 synergies, decreasing $Allow_{a}$ from 1.00 to 0.50 produced the highest correlation for ES R and a comparable correlation for ES L. Overall, 7 synergies and the $Allow_{a}$ = 0.50 provided the most accurate estimation (Table 3; Figure 4). The correlation between estimated ES activations using the SYN method with seven synergies and the measured ES muscle excitations was greater (>0.15), compared to estimates using the static optimization method (Table 3; Figure 4).

The joint moment matching performance of SYN was dependent upon both the number of muscle synergies and the $Allow_{a}$ value in the optimization objective function. As the number of synergies used for estimation increased from 5 to 8, the mean RMSE of the estimated joint moments decreased, indicating that the performance of moment matching improved (Table 4). By increasing the number of muscle synergies, the number of design variables in the optimization, synergy vector weights, was also increased, allowing for greater flexibility to better track the experimental joint moments. For each number of synergies evaluated, reducing the $Allow_{a}$ value would increase the mean RMSE of the estimated joint moments in lumbosacral extension and bending while notably decreasing the mean RMSE in lumbosacral rotation (Table 4). Reducing $Allow_{a}$ would increase the weight of the activation minimization term relative to the moment tracking term in the optimization. It was therefore anticipated that the optimizer would reduce activations further at the expense of moment matching quality. Even though the mean RMSE for lumbosacral rotation was lower, it might not have been enough to offset the increase in the mean RMSEs for lumbosacral extension and bending because the optimizer minimized error in joint moment matching for all three DoFs collectively. Therefore, 8 synergies and $Allow_{a}$ = 1.00 provided the best overall performance for joint moment matching.

The two metrics used to evaluate the performance of SYN provided relatively different information regarding the optimal number of synergies and $Allow_{a}$ . Despite the fact that the mean RMSE of the estimated lumbosacral joint moments was lower with 8 muscle synergies than with 7 muscle synergies, the higher correlation between the measured and estimated muscle activity of ES with 7 muscle synergies should be viewed as more important given that the objective of the study was to estimate realistic trunk muscle activations. The more accurate estimates for a key trunk muscle might outweigh the small improvement (the difference in mean RMSE was within 1 Nm for all 3 lumbosacral DoFs) in matching the experimental joint moment with the 8 synergies. The $Allow_{a}$ of 0.5 was superior to the other values tested because of the highest overall correlation it produced with 7 synergies (Table 4; Figure 4). For the purposes of this study, seven muscle synergies and a value of 0.50 for $Allow_{a}$ were deemed the best-case scenario.

The desired activation estimation performance with seven synergies is probably due to the more uniform spacing between synergy activations throughout a gait cycle. Muscle synergy activation peaks are more likely to be distributed throughout the gait cycle as the number of synergies increases (Supplementary Figures S4A,B). This increases the likelihood that a muscle synergy emerges in unison with one or more trunk dynamical events. These distinct synergies would be recruited by the trunk muscles in an attempt to reproduce the dynamic events. For example, lumbosacral extension joint moments were required at the beginning and end of the gait cycle. Synergies 4 and 5 in the representative gait cycle had peak activations at those points (Supplementary Figure 5A). The two muscle synergies were both recruited by the lumbar extensors ES and MF. This observation was also consistent with the reported activation of ES during footstrikes (White and McNair, 2002). Due to the emergence of such synergies, trunk muscles no longer needed to recruit all the synergies made available to them by lower extremity muscles. We observed minimal recruitment of at least one synergy on each side of the body by the ipsilateral trunk muscles (Supplementary Figures S4A,B). This observation brings our result closer to the published data indicating that muscle activity during gait could be explained by five activation patterns (Ivanenko et al., 2004).

**FIGURE 5:**
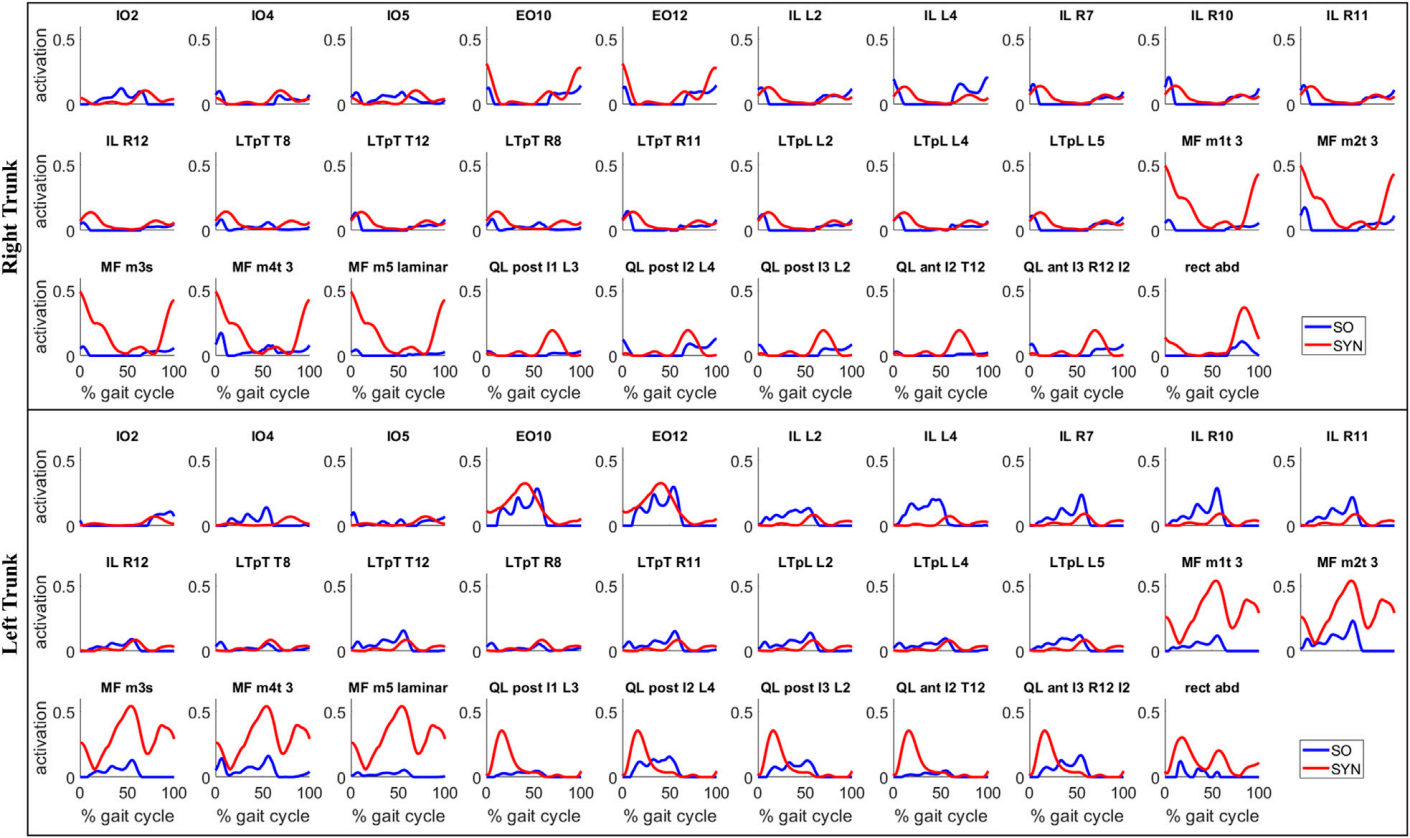
Estimates of trunk muscle activations by the proposed synergy-based method (SYN) and by the static optimization method (SO) for a representative gait cycle. For SYN, the activations were estimated with 7 muscle synergies per side and $Allow_{a}=0.50$ . The name above each subplot indicates an individual muscle head, and the larger muscle to which each muscle head is associated is listed in Table 2.

SYN demonstrated several advantages over SO in addition to its superior estimation of ES activations (Figure 4). Firstly, SYN underestimated activation levels for trunk muscles EO, ES, MF, and RA less frequently than SO. SO did not recruit these muscles during the majority of the ipsilateral leg’s stance phase (Figure 5), which may not be desirable for maintaining posture control and joint stability (Lee et al., 2006; Rosa et al., 2014). Secondly, the SYN estimates of trunk muscle activation were smooth curves with gradual changes, while the SO estimates permitted abrupt activation changes to match the experimental joint moments, which may not be physiological. SO estimated three consecutive activation peaks for muscles IO4 (L), EO10 (L), and EO12 (L) during the stance phase (Figure 5). This may be due to the nature of SO, which solves one frame at a time and sometimes provides little continuity between successive time frames (Shourijeh and Fregly, 2020). In contrast, SYN simultaneously solves all time frames, and muscle activation curves take on the smooth shapes typical of synergy activations. Thirdly, unlike SO, the structure of SYN causes muscle heads to have a similar pattern and magnitude. Despite belonging to the same muscle group (IO), SO activated IO2, IO4, and IO5 during different gait cycle phases (Figure 5). We compared SYN and SO because SO is commonly used to estimate muscle activation in the absence of muscle EMG measurement. Even though SO performed better than SYN in matching the experimental joint moment, SYN may be more suitable for estimating trunk muscle activations, which was set as the primary objective of this study.

Our proposed computational framework for estimating trunk muscle activations required a full-body musculoskeletal model that included lower extremity and lower trunk muscles (Figure 3). Though full-body OpenSim models with lower extremity and trunk muscles already existed (Raabe and Chaudhari, 2016; Schmid et al., 2020; Favier et al., 2021), we decided to create our own model based on M1 (Rajagopal et al., 2016) and M2 (Bruno et al., 2015) for the following reasons: First, both models would permit customization of muscle strength using the subject’s readily accessible height and mass data. Using such data from the subject, the volume and peak isometric force of the 35 lower extremity muscles in M1 can be calculated (Handsfield et al., 2014). ACSA of torso muscles, used to estimate the muscle peak isometric force parameter, could be estimated using regressions based on the gender, age, height, and mass of the subject (Anderson et al., 2012). Second, the EMG-driven lower extremity musculoskeletal model used in this study (Ao et al., 2022) was developed using the M1 model. Third, the lower extremity model developed to simulate high hip-knee flexion activity (Lai et al., 2017) was also based on M1. The pre-existing full-body OpenSim models mentioned above were not derived from M1 or M2. Therefore, we decided to combine the M1 and M2 musculoskeletal models to meet the requirements of our clinical application for pelvic sarcoma (Figure 1).

Model personalization improved the model’s ability to accurately represent the subject’s kinematics and kinetics in multiple ways, generating more subject-specific data for estimating trunk muscle activations. First, the image-based modeling approach provided a possibly more accurate representation of the pelvic region’s musculoskeletal system. The centers of the hip and lumbosacral joints were placed more precisely than a marker-based scaling method could have. The sacroiliac angle was personalized to be representative of the subject’s anatomy. Muscle attachments were placed at the appropriate locations based on the pelvic bone geometry of the subject. As a result, the subject-specific model produced more accurate results of joint kinematics, muscle-tendon lengths, and muscle moment arms, which were required for estimating trunk muscle activations and forces. The Hill-type muscle model parameters for the trunk muscles were also personalized. The majority of trunk muscles in the model gained strength (Table 2) and operated at more optimal lengths for force generation (Supplementary Table S1, Supplementary Figure S1). Without these model modifications, trunk activations of much greater magnitude would be expected to generate joint moments. Lastly, the performance of SYN depended heavily on the synergies extracted from the activations of the muscles of the lower extremities. Our comprehensive EMG-driven model (Ao et al., 2022) provided reliable estimates of lower extremity muscle activations (moment mean absolute error 5.5 Nm) using measured muscle excitations while predicting unmeasured muscle excitations (Supplementary Figure S2).

This study has several major limitations due to its definite scope. First, this study only included data from a single subject, meaning that the findings may not be applicable to other subjects with comparable or dissimilar pathology. Nonetheless, it is encouraging to see that the essential methodological components, such as a suitable model, model personalization techniques, and computational algorithm, have been developed and can facilitate the analysis of data collected from future subjects recruited. Due to the scarcity of available datasets and the difficulty in recruiting more patients and acquiring a dataset of this extent, this study is limited to data from a single subject. This study’s extensive dataset included medical imaging and a large number of EMG recordings from the leg and trunk, including fine wire recordings of deep muscles and motion and ground reaction data for multiple gait cycles. The availability of such a dataset represents a rare opportunity to develop a subject-specific musculoskeletal model. Second, only one task’s movement data was analyzed. Future research could expand on the current study by incorporating additional daily activities, such as squatting and stair ascending and descending. Third, our trunk musculoskeletal model is simplified. The spine is partially articulated, with joints at L5-S1 and T8-T9, and is actuated by a reduced set of muscles. The reason for the simplification is that the large number of body segments, joint degrees of freedom, and muscles in a more detailed lumbar spine model would likely complicate model-based motion predictions, which is our ultimate research objective. Due to the limited range of motion of the spine during gait, we believe the partially articulated spine model is justifiable for at least gait motion. Fourth, we used non-negative matrix factorization (NMF) to decompose the lower extremity muscle activations for synergy extraction, as it provides the most accurate reconstruction of measured muscle activations or excitations during walking compared to other matrix factorization algorithms (Rabbi et al., 2020), and non-negative synergy activations and synergy vector weights were deemed more physiological. However, NMF is frequently unable to provide a unique solution (Shourijeh et al., 2016), which may introduce uncertainty into our computational method. As we discovered that the quality of our estimation was dependent on the extracted synergies, it would be advantageous to consider alternative factorization techniques, such as principal component analysis, independent component analysis, and factor analysis, for future research. Fifth, we have validated our estimation of trunk muscle activations using only experimental measurements for one trunk muscle, ES. Because ES is one of the most studied trunk muscles (van Dieën et al., 2003) and one of the primary agonists for lumbar extension and lateral bending, it was selected for EMG measurement. If, in the future, additional EMG channels become available for recording, we will measure the activity of other trunk muscles for additional validation.

In conclusion, this study presented a novel computational method for estimating trunk muscle activations using muscle synergies extracted from lower extremity muscle activations. Preliminary studies performed on the gait data of a pelvic sarcoma patient have returned generally realistic estimates of trunk muscle activations, demonstrating the feasibility and effectiveness of the proposed method. The proposed method can be utilized to address a variety of research issues. From a practical standpoint, our proposed method can address a common problem when insufficient EMG channels prevent simultaneous measurement of trunk and lower extremity muscle activity. From the perspective of improving the standard of care for patients with pelvic sarcoma over the long term, the knowledge gained from the activation and force of trunk muscles can be useful for a number of clinical applications involving pelvic sarcoma. One of the primary research objectives of the authors is to use the activations and synergy structures identified in this study to develop subject-specific synergy-driven models (Meyer et al., 2016; Sauder et al., 2019) of pelvic sarcoma patients in order to predict postoperative functional outcomes. The prediction will be used to evaluate and optimize surgical treatment designs in order to maximize functional outcomes following surgery. The other primary objective of the authors’ research is to develop load cases for stress analysis of custom prosthesis designs using muscle force estimates, which may enhance the performance and durability of such designs.

## Data Availability

The experimental data, OpenSim models, and Matlab code used to perform this study are available at https://simtk.org/projects/synesttrunkact/.
