# Supplementary material for: A computational method for estimating trunk muscle activations during gait using lower extremity muscle synergies
Source: Front Bioeng Biotechnol. 2022 Dec 13;10:964359. doi: 10.3389/fbioe.2022.964359 (PMC9792665; doi:10.3389/fbioe.2022.964359)
Supplement: Supplementary file 1 [file DataSheet1.pdf]

# *Supplementary Material*

## 1 Static Optimization Formulation

The static optimization method was formulated as a quadratic programming problem, which was solved using MATLAB "quadprog" function, as follows:

$$\min_{a_j} \sum_{j=1}^{nMusc} a_j^2$$

subject to

$$M_i = \sum_{j=1}^{nMusc} r_{ij} F_j$$

$$0 \leq a_j \leq 1$$

where $a_j$ is the activation of the $j^{th}$ muscle $j = 1, \ldots, nMusc$, and $nMusc$ is the number of trunk muscles. $M_i$ is the inverse dynamic joint moment of the $i^{th}$ lumbosacral joint DoF excluding psoas group contribution. $i = 1,2,3$ for lumbosacral extension, bending, and rotation, respectively.

## 2 Supplementary Data

**Supplementary Table S1**. The mean normalized muscle fiber lengths of trunk muscle groups. Scaled values were obtained using the tendon slack lengths and optimal fiber lengths in the scaled generic model. Adjusted values were obtained using the tendon slack lengths and optimal fiber lengths found through optimization (Section 2.3.3 of the manuscript).

| | Right Side | | | | | | Left Side | | | | | |
|---|---|---|---|---|---|---|---|---|---|---|---|---|
| | IO | EO | ES | MF | QL | RA | IO | EO | ES | MF | QL | RA |
| Scaled | 0.98 | 1.03 | 0.81 | 0.79 | 0.87 | 1.01 | 1.03 | 0.98 | 0.80 | 0.77 | 0.83 | 1.00 |
| Adjusted | 0.94 | 0.93 | 0.89 | 0.88 | 0.87 | 0.98 | 0.95 | 0.91 | 0.86 | 0.84 | 0.81 | 0.98 |

$$\tilde{l}^M = \frac{l^{MT} - l_s^T}{l_o^M \cos\alpha}$$

where $\tilde{l}^M$ is the normalized muscle fiber length, $l_o^M$ is the optimal fiber length, $l_s^T$ is the tendon slack length, and $\alpha$ is the pennation angle.

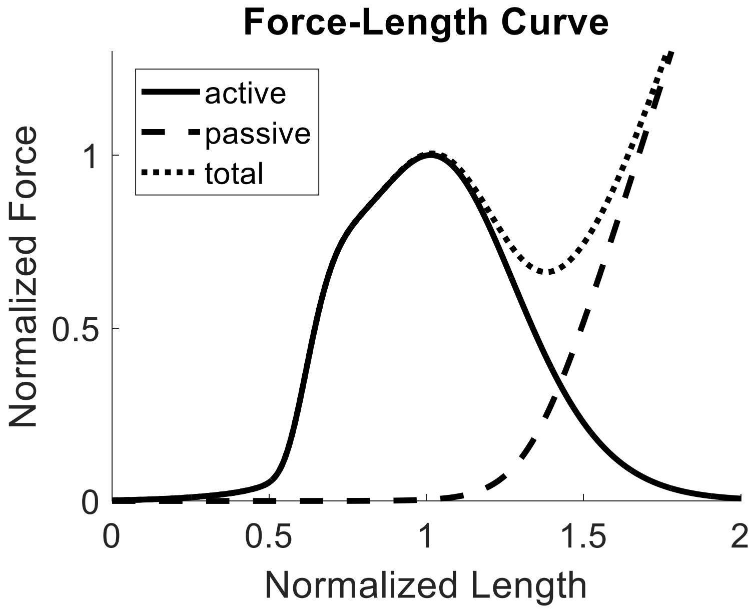


**Supplementary Figure S1.** Normalized muscle force-length curves. On the ascending region of the total force when normalized length is slightly less than 1, the active force generated is close to the normalized peak isometric force whereas the passive force generated is nearly zero.

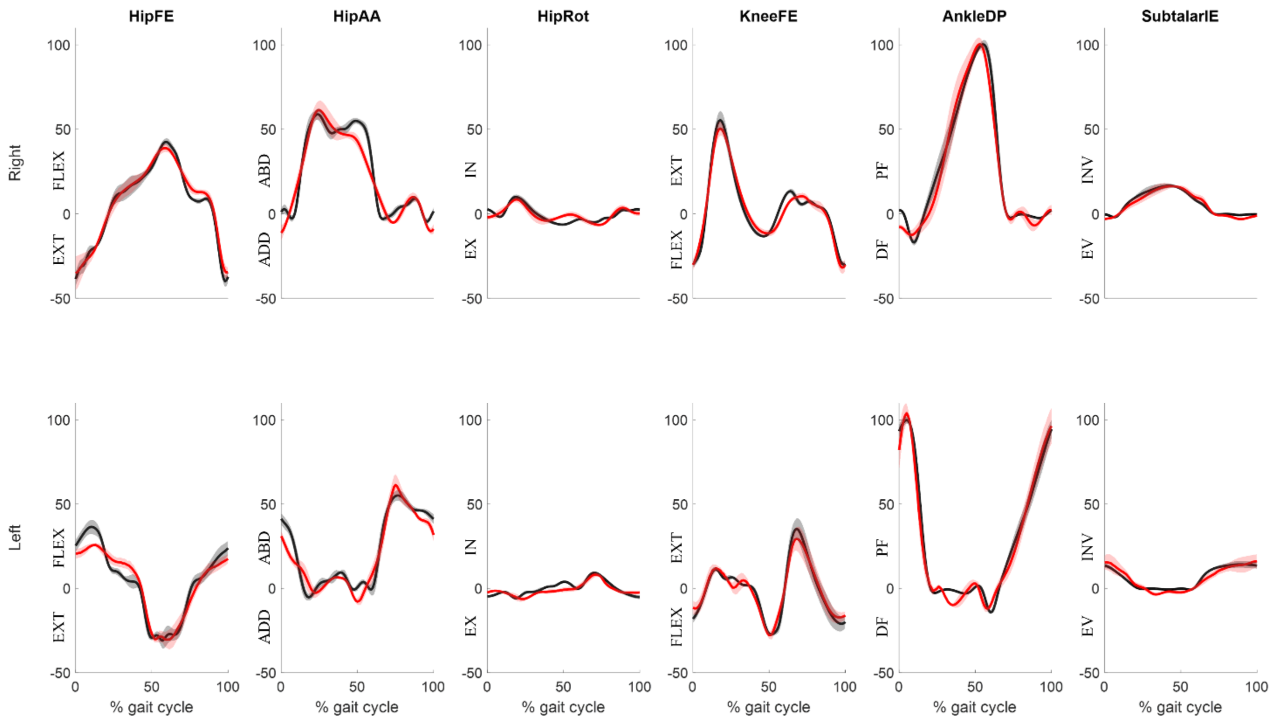


| | Mean Absolute Error in Joint Moment Estimates (Nm) | | | | | |
|---|---|---|---|---|---|---|
| | HipFE | HipAA | HipRot | KneeFE | AnkleDP | SubtalarIE |
| R | 4.9 | 4.7 | 2.5 | 5.5 | 4.8 | 2.9 |
| L | 4.8 | 4.5 | 1.8 | 2.5 | 4.0 | 1.7 |

**Supplementary Figure S2.** Joint moments estimated from the EMG-driven model of the lower extremity. Solid line: mean of joint moments, the shaded region: ±1 standard deviation of joint moments. Black: inverse dynamics, Red: EMG-driven estimates. FLEX – flexion, EXT – extension, ADD – adduction, ABD – abduction, EX – external, IN – internal, DF – dorsiflexion, PF – plantarflexion, EV – eversion, IV – inversion.

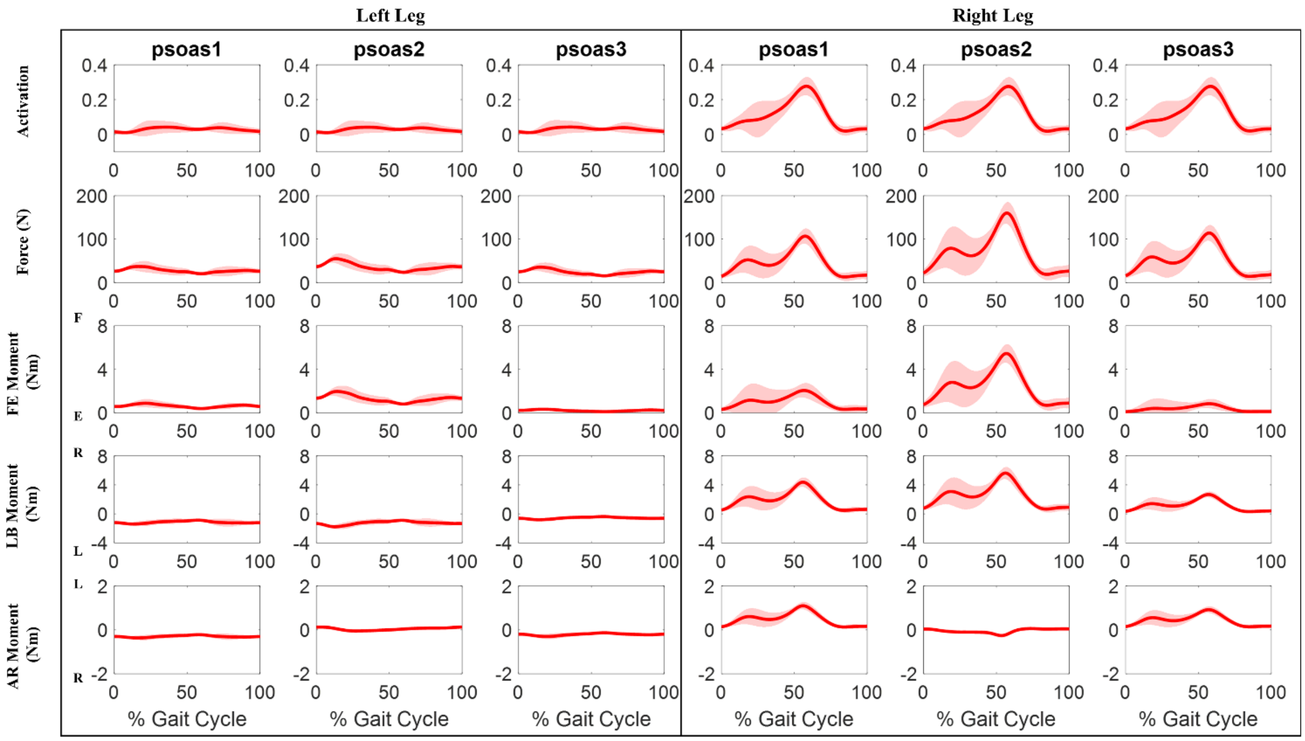


**Supplementary Figure S3.** Activations, forces, and lumbosacral joint moments of psoas muscles estimated by the EMG-driven model. FE – flexion (+, F) or extension (–, E). LB – lateral bending toward right (+, R) or left (–, L). AR – axial rotation toward left (+, L) or right (–, R).

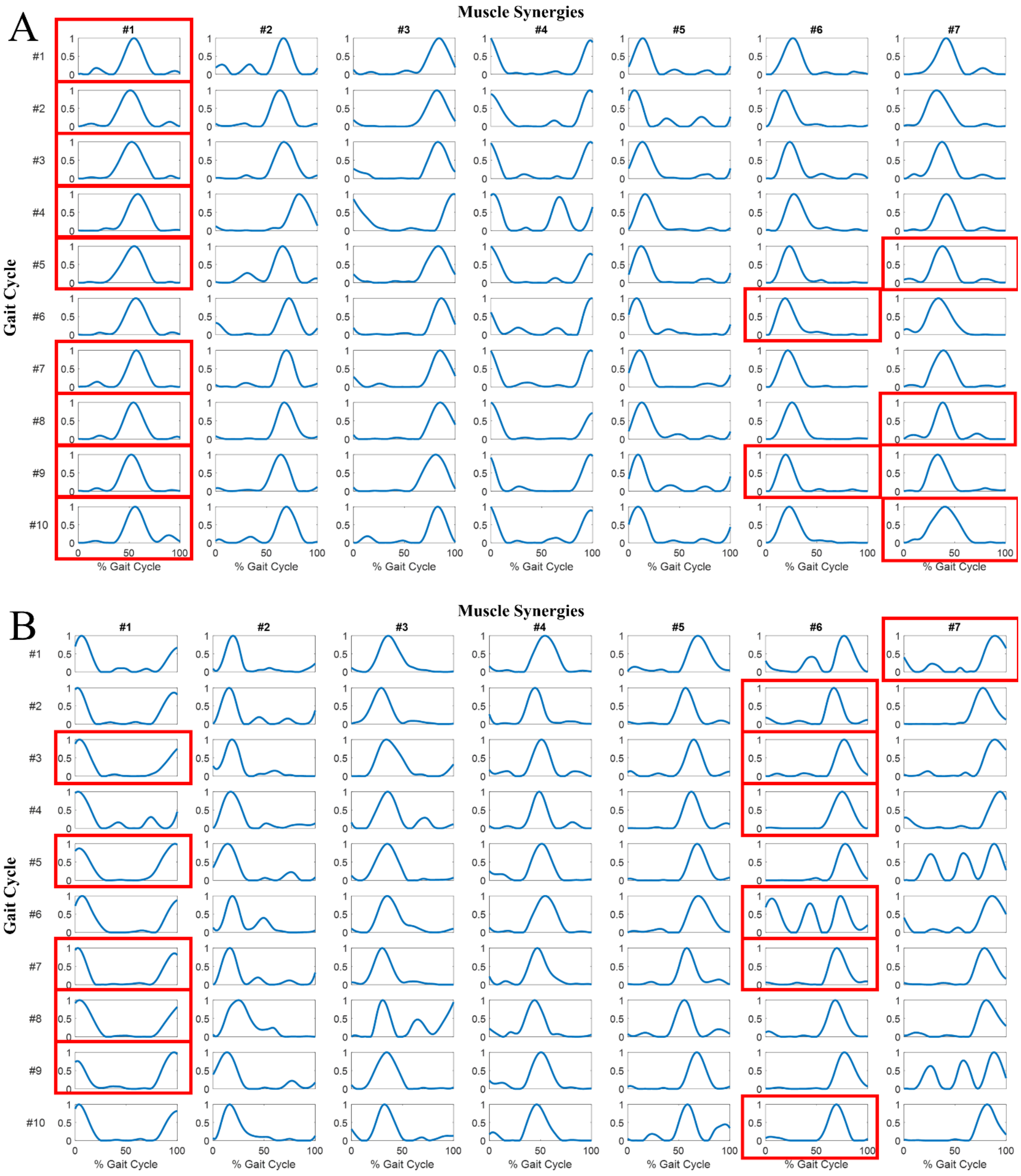


**Supplementary Figure S4.** The synergy activations of the 7 muscle synergies extracted from **(A)** the right leg muscles, and **(B)** the left leg muscles, for each of the 10 gait cycles that were analyzed. Red boxes highlight the synergy activations that were minimally recruited (synergy vector weights < 0.01) by the ipsilateral trunk muscles.

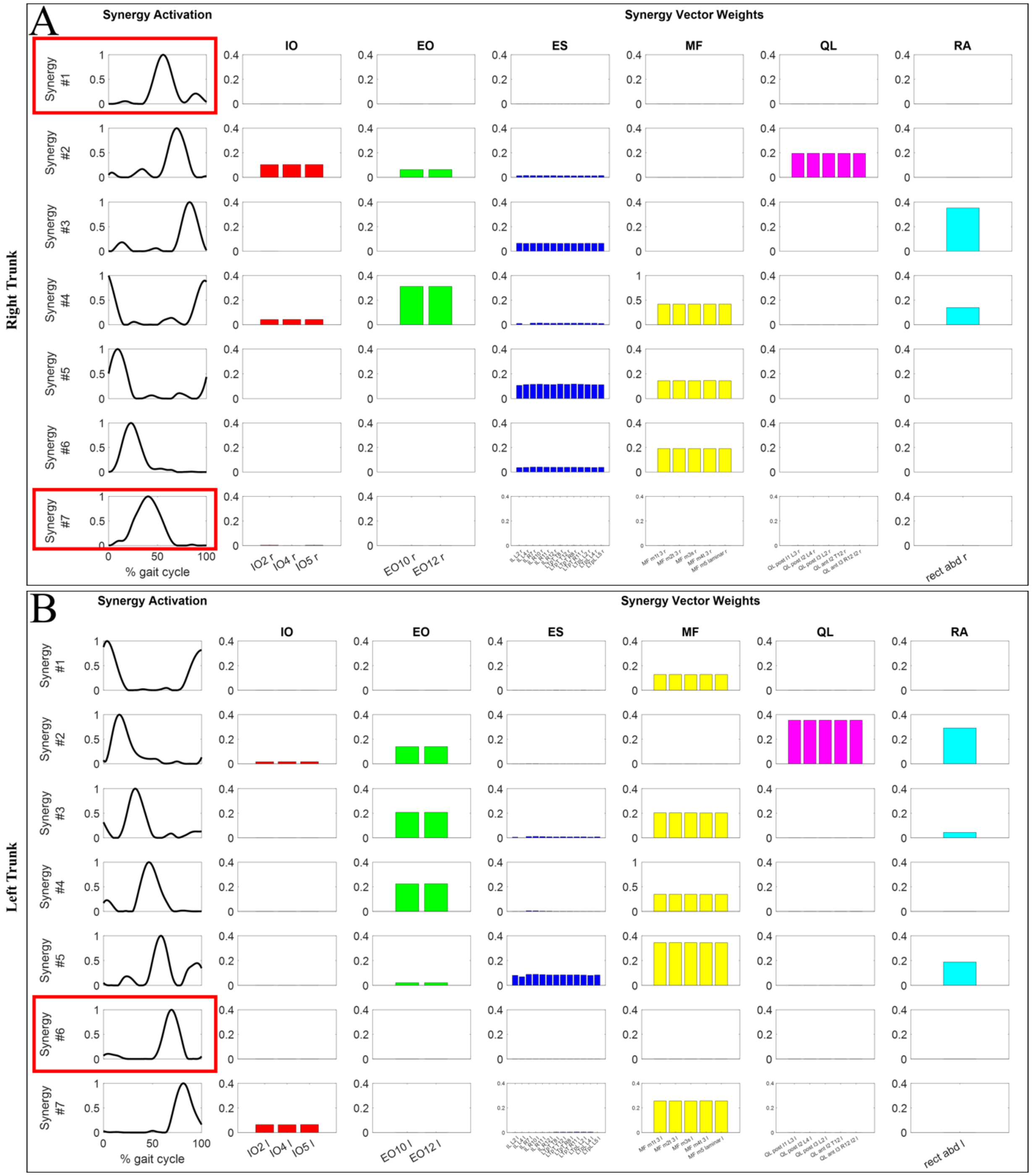


**Supplementary Figure S5.** Synergy components for **(A)** the right trunk muscles and **(B)** the left trunk muscles during a representative gait cycle. A synergy activation in a red box indicated that trunk muscles did not recruit the synergy. $Allow_a$ = 0.50 was used in the optimization.
